# Oral micro-electronic platform for temperature and humidity monitoring

**DOI:** 10.1038/s41598-023-48379-9

**Published:** 2023-12-02

**Authors:** Željko V. Popović, Aung Thiha, Fatimah Ibrahim, Bojan B. Petrović, Nuraina Anisa Dahlan, Lazar Milić, Sanja Kojić, Goran M. Stojanović

**Affiliations:** 1 Faculty of Technical Sciences, University of Novi Sad, Trg Dositeja Obradovica 6, 21000 Novi Sad, Serbia; 2 Department of Biomedical Engineering, Faculty of Engineering, Universiti Malaya, 50603 Kuala Lumpur, Malaysia; 3 Centre for Innovation in Medical Engineering, Faculty of Engineering, Universiti Malaya, 50603 Kuala Lumpur, Malaysia; 4 Microwave Research Institute, Universiti Teknologi MARA, 40450 Shah Alam, Malaysia; 5 Faculty of Medicine, University of Novi Sad, Hajduk Veljkova 3, 21000 Novi Sad, Serbia

**Keywords:** Engineering, Biomedical engineering, Electrical and electronic engineering, Saliva

## Abstract

Intraoral theranostics, the integration of diagnostics and therapeutics within the oral cavity, is gaining significant traction. This pioneering approach primarily addresses issues like xerostomia (dry mouth), commonly resulting from cancer treatment, with a specific focus on monitoring temperature and humidity. This paper introduces the innovative Intra-Oral Portable Micro-Electronic (IOPM) fluidic theranostic device platform. It leverages conventional dental spoons by incorporating advanced sensors for precise measurements of oral temperature and humidity. Personalization options include a microfluidic chip and a tooth model, enabling targeted delivery of therapeutic agents to optimize treatment outcomes. The electronic control system simplifies the administration of fluid dosages, intelligently adjusted based on real-time oral cavity temperature and humidity readings. Rigorous experimental evaluations validate the platform’s precision in delivering fluid volumes at predefined intervals. This platform represents a transformative advancement for individuals contending with oral health challenges such as xerostomia (dry mouth). Furthermore, it has the potential to elevate oral healthcare standards by providing advanced diagnostics and tailored therapeutic solutions, benefiting both patients and dental professionals alike.

## Introduction

Oral diseases represent a significant global health challenge, impacting millions of individuals worldwide. Timely and precise screening for these conditions is critical for early detection, effective treatment, and overall oral health improvement. In recent years, there has been a concerted effort to develop state-of-the-art techniques for screening oral diseases, with a specific emphasis on screening for oral cancer. The brief standard procedures for screening oral cancer are as following:

- *Visual and clinical examination* Visual and clinical examination by trained healthcare professionals remains the cornerstone of oral cancer screening. Dentists and oral healthcare providers conduct thorough examinations of the oral cavity, looking for suspicious lesions, discolorations, and irregularities. Specialized tools like toluidine blue staining or autofluorescence devices aid in identifying potentially malignant or cancerous lesions. Visual examination, although non-invasive, heavily relies on the clinician’s expertise and may have limitations in detecting early-stage lesions^1^.
- *Tissue biopsy and histopathology* When suspicious lesions are identified, tissue biopsy followed by histopathological examination is the gold standard for confirming oral cancer diagnosis. Biopsies provide essential information about the nature and extent of the lesion, aiding in treatment planning. However, this approach is invasive and requires specialized expertise^2^.
- *Advanced imaging techniques* Advanced imaging techniques such as positron emission tomography-computed tomography (PET-CT) and magnetic resonance imaging (MRI) play a vital role in staging oral cancer and assessing its spread to surrounding tissues and lymph nodes. These modalities provide a comprehensive view of the disease’s extent, assisting in treatment planning.

Worrisome trends of oral cancer mortalities by geographical location call for effective diagnosis, treatment, and monitoring approaches to countermeasure the current 5-year survival rate of ≤ 50%. Oral cancer development is majorly contributed by the formation of malignant neoplasms in the oral mucosa. Globally, oral cancer reported 354,864 new cases and 177,384 deaths in 2018 alone. Seventy percent (70%) of the reported oral cancer deaths occurred in Asia due to the unregulated use of tobacco products, especially in chewable forms^3,4^. To date, oral cancer accounts for less than 5% of all cancer cases worldwide. Hence, it is often overlooked in the development of public health policies due to limited data on trends related to morbidity, mortality, and survival rate. Nevertheless, oral cancer treatment costs have been imposing a heavy economic burden on both low- and middle-income families as well as healthcare systems in most affected countries such as India, Bangladesh, Sri Lanka, and Pakistan^3,5^. Despite the advancement in medical diagnostic, post-operative care, and cancer therapy (e.g., radiotherapy, chemotherapy), relatively high numbers of patients are experiencing oral cancer recurrence. Furthermore, the adverse effects of cancer treatment on oral health, particularly the development of dry mouth or xerostomia due to damaged salivary glands, have gained significant attention. Cancer treatments, including radiation therapy and chemotherapy, often have adverse effects on oral health, leading to conditions like dry mouth (xerostomia). Xerostomia can result in discomfort, difficulty in eating and speaking, and an increased risk of oral infections and dental caries.

Several observational studies reported a high prevalence rate of dry mouth among cancer patients ranging between 40 and 83%. Indeed, an early cancer diagnosis improves the prognosis and quality of treatments while minimizing healthcare expenditures. Monitoring the temperature and humidity in the oral cavity is crucial in understanding and managing this condition^6^.

Temperature fluctuations in the oral cavity during physiological activities and consumption of hot and cold liquids have been extensively studied. Airoldi et al. found changes in temperature ranging from 10 to 55 °C after drinking hot and cold beverages^7^. Youngson and Barclay reported a maximum temperature of 68.0 °C and a minimum temperature of 15.4 °C in the oral cavity^8^. Temperature variations were less extreme in the back regions of the mouth. Palmer et al. used a digital thermometer and found a temperature range of 0 °C to 67 °C^9^. These studies provide valuable insights into temperature variations in the oral environment.

State-of-the-art biosensor technologies are highly regarded as effective portable devices that combine molecular biology, microfluidic, system integration, and data science for rapid monitoring and therapy of oral cancer^10^. The automated system has the capability to sense, mix, pump, and control fluidic volumes at predetermined conditions such as moisture level, temperature, pH, and electrolyte compositions^6,11^. Measuring body temperature is a crucial aspect of the diagnostic technique. A slight difference in body temperature can be a great indicator to detect warning signs of infections^12^. Analyzing intra-oral temperature is important to evaluate patient status in dentistry and medicine. Sublingual temperature is related to the core temperature and can reflect changes in body temperature that may indicate the onset of infections, reactions to medications, or other disease-specific symptoms^13,14^. In dentistry, intra-oral temperature can be a valuable diagnostic tool for detecting various oral problems. Saliva analysis has shown promising results in detecting squamous oral carcinoma, particularly p53 as a biomarker^15^.

A dry mouth or xerostomia is a subjective feeling of oral dryness due to the reduced production of mucin in saliva. Dry mouth is a common problem among older people, people with Sjögren’s syndrome, and cancer patients. In cancer patients, the oral dryness condition is contributed by systemic conditions and side effects of cancer treatments, especially radiotherapy and chemotherapy^16,17^. A pioneering study by Shimosato et al*.*^17^ reported that advanced cancer patients’ self-reports of dry mouth might not be an accurate assessment and diagnosis of oral dryness. In this study, oral dryness symptoms measured using an oral moisture checking device by experienced dentists reported that 21.4% of patients diagnosed with xerostomia did not self-report any oral dryness symptoms. Their findings demonstrated the importance of an accurate xerostomia diagnosis for effective palliative oral care. Bots et al*.* conducted a study with the goal of visually detecting xerostomia through photography, which led to a negative result. It is stressed that dentists are as good as non-dentists in judging pictures of the tongue, as a method for the diagnosis of xerostomia^18^. Recently, Karthikeyan et al*.*^19^ reported a new dental technique to detect dry mouth by integrating a pressure sensor within a dental prosthesis. The system was equipped with a reservoir containing artificial saliva for immediate treatment of oral dryness in edentulous patients. Future testing of the sensor can be used as a platform for dental prosthesis monitoring. Despite the promising potential, their dental implantable pressure sensor lacks some core aspects, including issues with manufacturing the device, bulky sensors, standardisation of accurate saliva injections, biocompatibility, and durability concerns. Up to now, a salivary-based sensor for managing xerostomia is still in its infancy, with many potentials yet to be explored for its effective implementation.

Recent efforts have led to significant development of intraoral electronic or wearable biosensors to detect biochemical markers for various real-time health monitoring^20–22^. Significant research progress on portable oral biosensors has transitioned to cost-effective monitoring using human biofluids such as blood, sweat, and tears. Saliva has risen to fame of late as a reliable monitoring tool owing to its non-invasive and low-cost characteristics^23^. Saliva offers an alternative to conventional blood analysis, therefore, eliminating the risk of infections, pain, and stress caused by invasive blood sampling^17^. Despite the increasing popularity of salivary-based biosensors, the forefront challenge of successful portable theranostic devices is a lower concentration of analytes in saliva (100 to 1000 lower) compared to whole blood samples. In addition, analyte compositions and overall saliva concentrations can be influenced by variations in the diurnal/circadian cycles. Therefore, the use of highly sensitive detection systems or technologies is warranted to circumvent the aforementioned limitations^24^. Since the invention of mouth-based portable electronic devices in the 1960s, research has moved towards integrating miniaturised electronic devices from voltage regulators to Bluetooth low-energy transceivers^25^. Recent research shows the profound advantages of portable electronic devices on an oral platform with high sensitivity and reliability using saliva^26,27^. For example, Arakawa et al*.*^28^ reported that the integration of a glucose sensor and wireless measurement system into a customized mouthguard showed a wide sensitivity range of 5–1000 μmol/L. A handful of studies reported on wearable salivary biosensors by integrating enzymatic electrodes on a mouthguard targeting fitness (e.g., lactate and uric acid monitoring) and biomedical applications^13,14^. Moreover, biosensors for lactic acid, uric acid and glucose have been already integrated in a mouthguard with all the following microelectronics integrated on the inside of the mouthguard^21,29^. Based on the discussed literature, several strategies have been established hitherto to develop oral biosensing platforms for health monitoring and biomedical applications. However, the integration of electronic sensing to portable devices for intra-oral monitoring and delivering therapeutic drugs post-oral cancer therapy is scarcely studied.

To our knowledge, we are the first to report a proof-of-concept of an intra-oral portable micro-electronic (IOPM) theranostics device with humidity and temperature sensing functions for intra-oral monitoring and therapeutics. Patients undergoing cancer treatments such as chemotherapy may experience elevated body temperature that could be a sign of fever or infections beyond the normal body temperature of 37 °C. Patients may also experience dry mouth owing to the temporary damage of salivary glands and other mouth-related side effects such as ulcers and mouth sores post-chemotherapy^30^. In this framework, the universal device has built-in sensors to detect changes in humidity and temperature in the oral cavity. Therefore, our study aims to detect dry mouth symptoms at humidity lower than 65% with elevated oral temperature by triggering localized delivery of artificial saliva for immediate treatments to alleviate side effects of post-chemotherapy. The multi-stimuli responsive sensors of the portable device are strategically designed to position precisely on the tongue, left, and right cheeks to ensure an accurate assessment of oral conditions followed by the release of therapeutic drugs in a predetermined pattern. In this study, 1 mL of artificial saliva will be immediately released for a duration of 3 s upon the influence of target stimuli. The IOPM device was constructed using dentistry-approved materials for effective implementation to the end users. Prospects of the fabricated portable device for intra-oral monitoring will be discussed with consideration of current and future challenges.

## Materials and methods

### Conceptual design of intra-oral portable micro-electronic (IOPM) theranostic device

**Figure 1:**
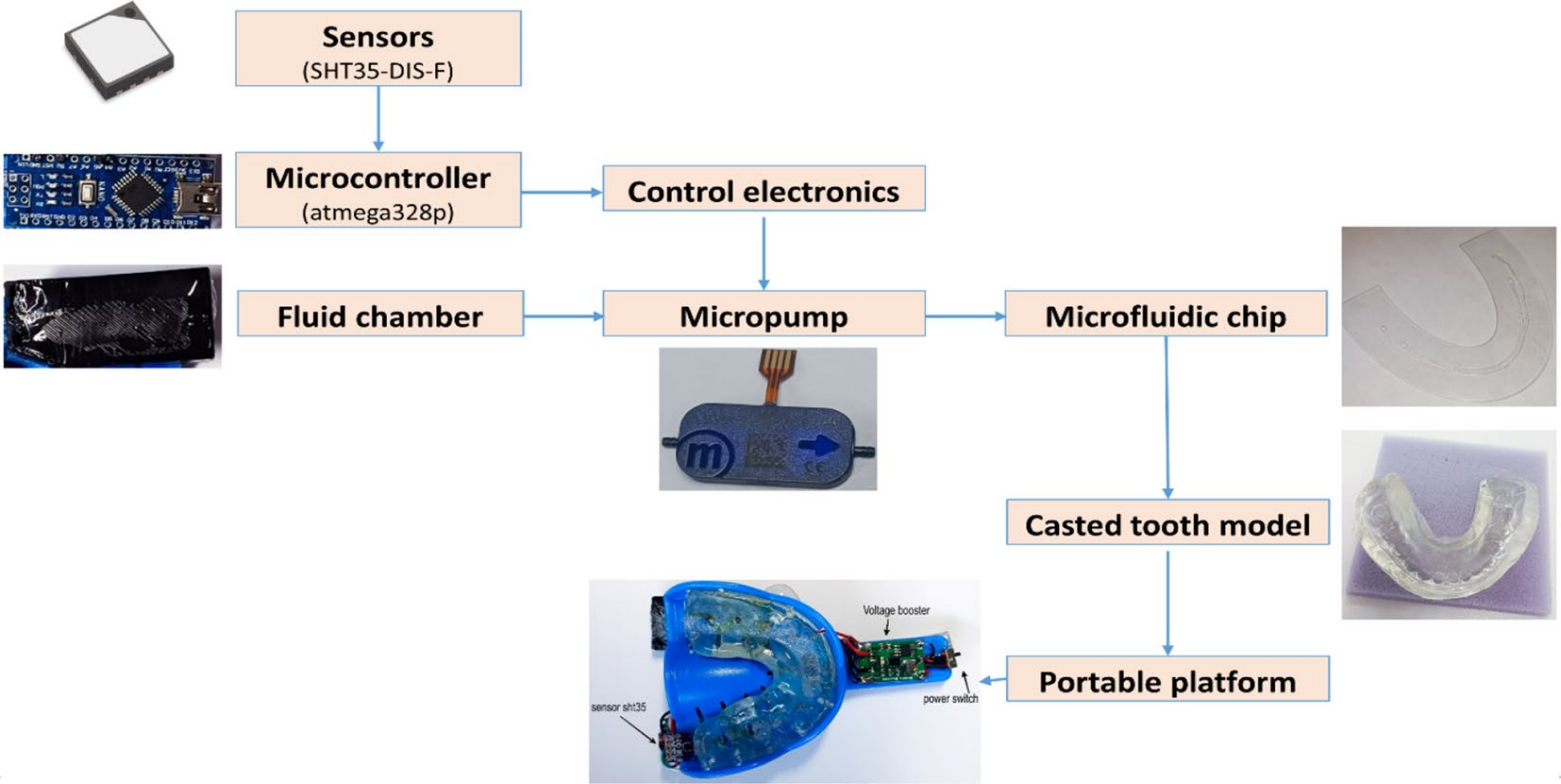
The conceptual design of the proposed platform of the intra-oral portable micro-electronic (IOPM) theranostic device.

The intra-oral portable micro-electronic (IOPM) theranostic device composed of hardware and software parts. Figure 1 illustrates the block diagram of the hardware part, which consisted of sensors, a microcontroller, a micro pump, and a microfluidic chip, as described in the following subsections.

### Design and fabrication of intraoral device

The basis for the intraoral appliance represents the dental impression tray, which was used to make dental anatomical model. This material was fully compatible for use in the oral cavity. The model of artificial teeth was made from acrylic resin. First, the tooth model was put into a dental impression spoon to take an impression using a clear vinyl polysiloxane material (EXACLEAR from GC EUROPE N.V.). The material has shown proven optimal consistency for the application in this study. The material was rigid and strong after setting in and easy to cut and drill injection holes. The casted dental model placed into the holder of the dentistry spoon was presented in Fig. 1.

### Sensors

To demonstrate the feasibility of intra-oral sensing, we integrated temperature and humidity sensors into the envisioned IOPM device. A commercial sensor (SHT35-DIS-F)^31^ was used to detect humidity and temperature inside the mouth precisely. To safeguard the sensor against environmental challenges such as water and dust, a PTFE membrane foil was strategically placed over it, adhering to IP67 standards. This ensured its functionality even in harsh environmental conditions. These sensors were meticulously calibrated, linearized, and equipped with temperature compensation for a digital output. Additionally, they featured an I2C interface capable of communication speeds up to 1 MHz, a crucial aspect for the proposed application as it allowed for rapid response when critical thresholds were met. The SHT35-DIS-F sensor boasts a typical accuracy level of 1.5% relative humidity and 0.1 °C. Nonetheless, the concept presented in this study provides flexibility, accommodating the use of any other commercially available or in-house developed sensor that is pertinent to oral cavity applications.

### Micropump

To facilitate the dispensing of the desired fluid, a specialized micropump was selected to meet precise criteria, which encompassed compact size, minimal weight, and a high degree of durability and dependability. The specific micropump, referred to as mp6, and its accompanying control electronics, denoted as mp-Lowdriver, were procured from Bartels Mikrotechnik GmbH in Dortmund, Germany^32^. The micropump technology employed in this system hinged on a piezoelectric diaphragm coupled with passive check valves. This mechanism involved a piezo ceramic component situated on a coated brass membrane, which underwent deformation when an electrical voltage was applied. This deformation action effectively facilitated the controlled release of the fluid from within the pump chamber.

### Microfluidic chip fabrication

**Figure 2:**
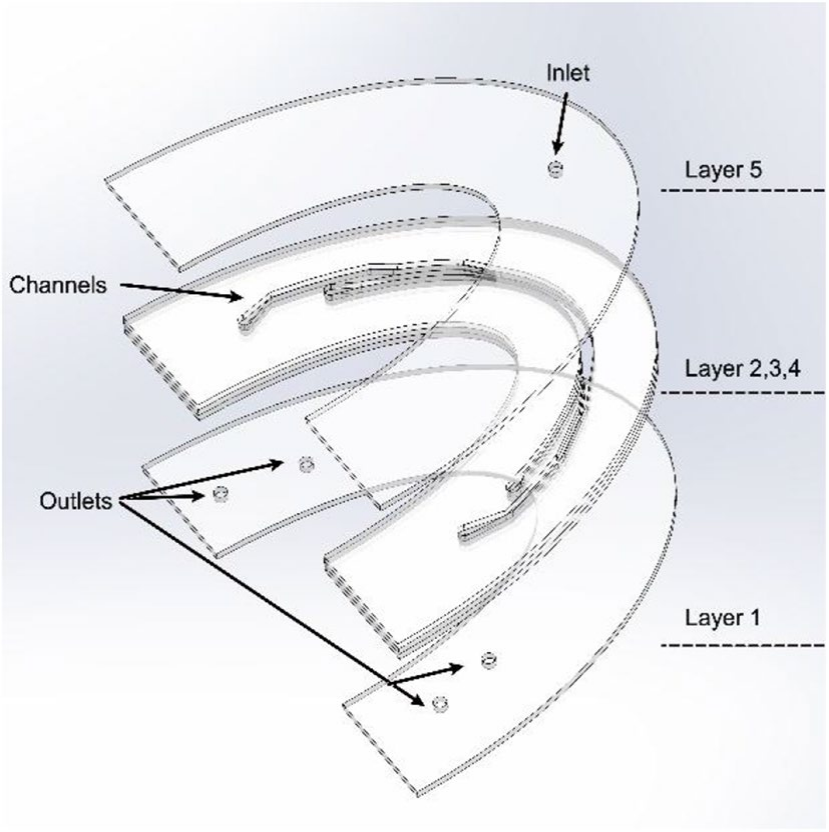
3D design of in-house developed microfluidic chip for personalized fluid delivery.

A microfluidic chip was designed and fabricated using xurographic technique^33^, with the primary goal of achieving precise fluid delivery to a specific tooth within the oral cavity. The chip was fabricated using a transparent polyvinyl chloride (PVC) A4 hot lamination foil with a thickness of 80 μm. The fabricating procedure was rapid (15 min in total with design), and the time required to create a single microfluidic chip using this technology in a laboratory environment was under 1 min. The proposed microfluidic chip consisted of five layers as depicted in Fig. 2. All layers were realized using PVC foils. The inlets and outlet of the microfluidic channels were cut using plotter cutting of PVC layers. In the final stage, lamination of the cut layers was performed^33^. The final product consisted of microfluidic channels with the following dimensions: 2 mm in width, 10 cm in length, and thickness of around 80 μm, while the inlet and outlet holes had a diameter of 2 mm. The shape and outer dimensions of the microfluidic chip were tailored to the dimensions of the standard dentistry spoon holder. This designed feature allowed for personalization or replacement of microfluidic chips based on the patient’s need or targeted tooth requiring fluid delivery.

### Software development of the IOPM theranostic device

**Figure 3:**
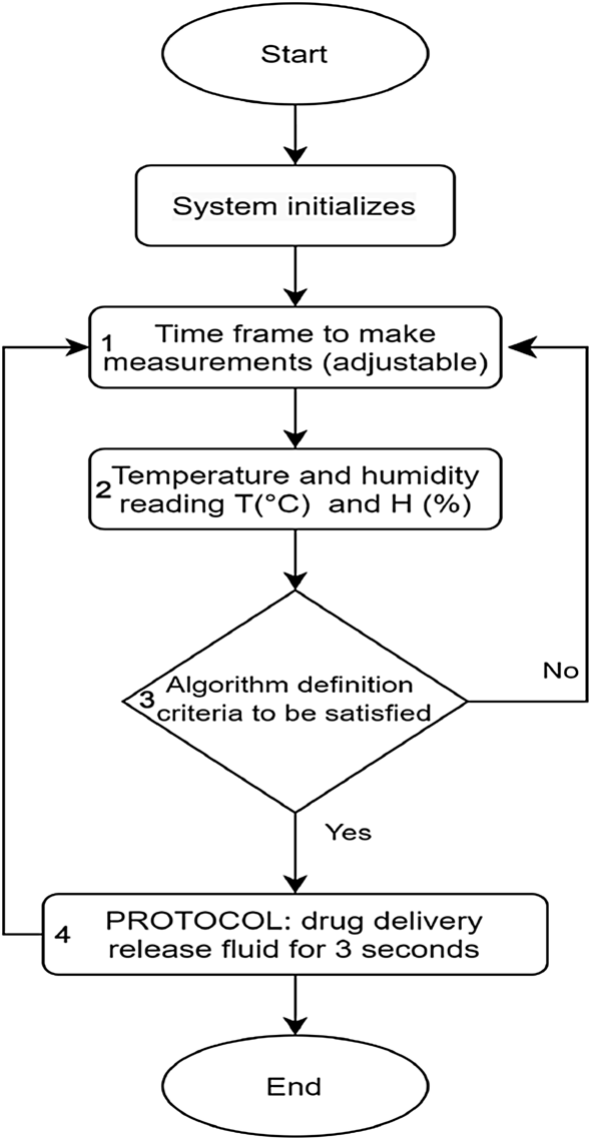
Flow chart of the software part of the IOPM platform.

The IOPM device is governed by software with the operational steps detailed in Fig. 3. The software begins when the system initialization button is pressed. At this point, both the temperature and humidity sensors are activated into action, commencing data sampling every 100 ms (indicated as number 1 in the flowchart).

The duration of this sampling timeframe can be adjusted within the software based on specific drug application requirements. For this experiment, we have chosen two critical values that are typically applicable in dentistry, as discussed earlier. In this particular setup, the criteria are set for the temperature to exceed 37 °C or for humidity to drop below 65% (marked as number 3 in the flowchart). Once one of these thresholds is met, the micropump is triggered to release 1 mL of fluid from the small reservoir for a period of 3 s (labeled as number 4 in the flowchart).

However, if the temperature remains below 37 °C or the humidity stays above 65%, the sensors will persistently monitor these two parameters until the criteria are eventually met.

### IOPM device functionality testing

**Figure 4:**
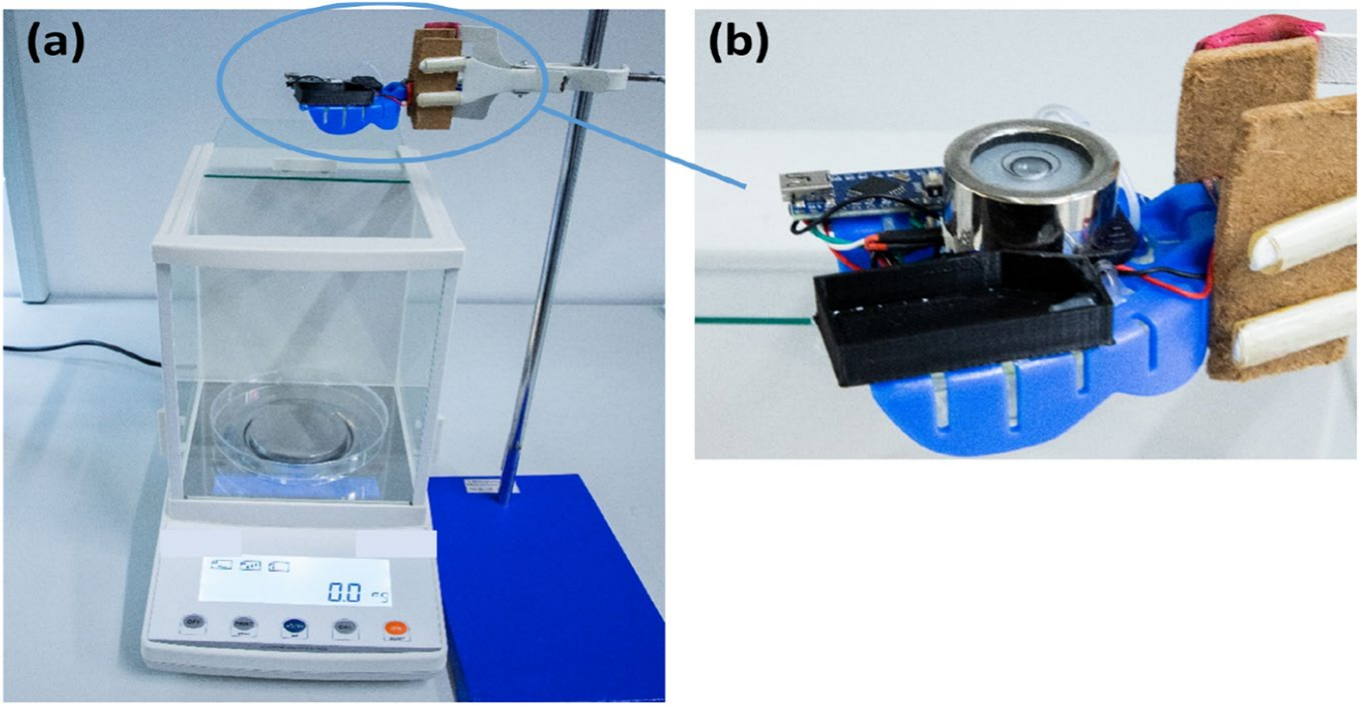
Experimental setup for (**a**) measuring released fluid, (**b**) horizontal device calibration.

The IOPM system was firmly positioned and set in a standardized configuration, as depicted in Fig. 4a, before conducting the experiments. To ensure a secure setup, a vertical stand was utilized to anchor the IOPM device, as illustrated in Fig. 4b. Accurate measurements of the dispensed fluid were obtained using an electronic analytical balance equipped with a high-precision electromagnetic balance sensor, capable of measuring with an accuracy of up to 0.0001 g (model: ANB-SIRET 220, COLO LabExperts, Slovenia). The IOPM device was programmed to activate the micropump for a duration of 7.5 s, resulting in the release of 1 mL of fluid as specified in the micropump's datasheet. This study tested two different liquids: distilled water (DI) and artificial saliva (AS).

During the test, the device was powered by a single 3.7 V Lithium Polymer battery cell. The experiments were repeated ten times following the same protocol. Briefly, the test system was emptied by operating the micropump on an empty fluid chamber, effectively pumping air into the system. The retainer was cleaned with an air shower and gently dried with paper tissues at the end of each cycle to eliminate any excess liquid. Subsequently, 5 mL of fluid was added to the fluid tank. The system was continuously operated until the first drop of the test solution burst out from the device’s output. Once the first droplet appeared, the device was turned off. Subsequently, the weight of the dispensed fluid droplets was gauged using a scale. Next, the weight of the released fluid droplets was measured using a weighing scale. Dead volume was calculated using Eq. (1). In this experiment, *Delivered liquid* referred to the measurements of the released fluid droplets during. *Inserted liquid* denoted the total volume of fluid present in the fluid chamber at the start of the experiments, while *liquid residuum* represented the remaining fluid in the fluid chamber after the experiments.

1 $$Dead \,volume= Inserted \,liquid - Delivered \,liquid - Liquid \,residuum.$$

System reaction time on temperature-dependent fluid dispensing was recorded using a high-speed camera (Canon 5d Mark IV). A heat gun was used to control the temperature of the simulated environment.

### Statistical analysis

Descriptive statistics were utilized to portray the data by displaying mean values and standard deviations. The normality of the data was assessed using the Shapiro–Wilk test. Regarding inferential statistics, the significance of distinctions among the researched groups was evaluated using the Wilcoxon rank-sum test. The statistical computations were conducted using the Statistical Package for Social Sciences (SPSS 20.0) in conjunction with Jamovi software (version 2.3.24)^34^.

## Results and discussions

**Figure 5:**
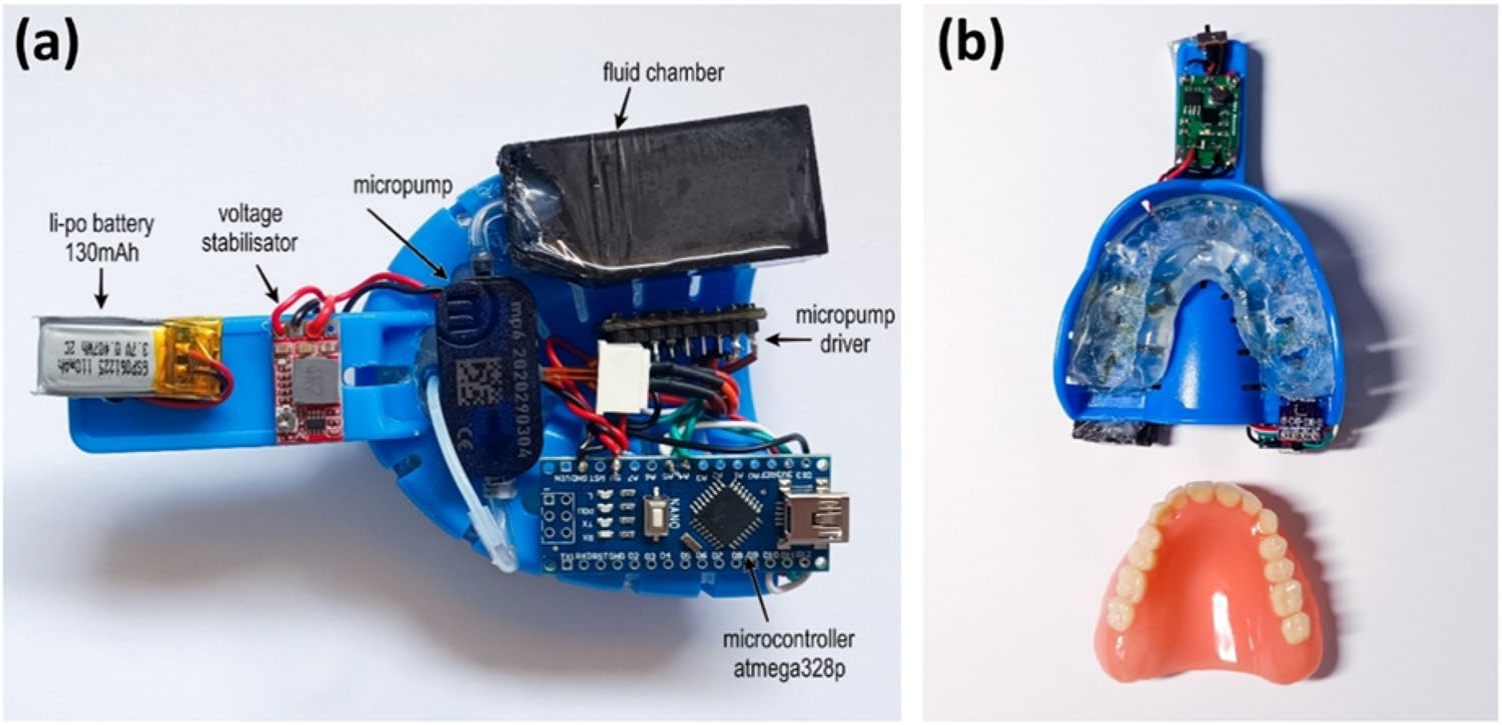
(**a**) The upper side of the developed IOPM prototype, (**b**) the side of the device which will be applied on the teeth (casted tooth model placed in the holder is also visible).

A custom-made intra-oral portable micro-electronic (IOPM) theranostic device was effectively designed and fabricated, as illustrated in Fig. 5. The central element of this IOPM theranostic device was the dental impression tray, typically employed in crafting a dental anatomical model.

The artificial tooth model was constructed using acrylic resin. Initially, the tooth model was positioned within a dental impression tray to create an impression using a transparent vinyl polysiloxane material^35^. This material became sturdy after setting, making it easy to cut and drill injection openings that corresponded to the outlets of the previously described microfluidic chip. Figure 5a displays the developed portable prototype of an electronic device for an intra-oral drug delivery system, featuring embedded sensors for monitoring the oral cavity's temperature and humidity. The molded dental model held within the dental impression tray is presented in Fig. 5b.

The complete IOPM prototype consisted of the following parts: a small transparent fluid chamber and a small lithium-polymer (LiPo) battery for powering the device. The battery was placed out the side making the final device wearable inside the mouth. When the battery was turned on, the original output of 3.7–4 V was low. Thus, a voltage booster was used to increase the voltage up to 5 V. The microcontroller atmega328p (Fig. 5a) was used to drive the system. One switch was placed to prevent the battery from powering the system for safety reasons. The bottom side of the device will have direct contact with the tooth and saliva. Therefore, a sensor SHT35 was placed in this location to measure the exact temperature and humidity in the oral cavity.

**Figure 6:**
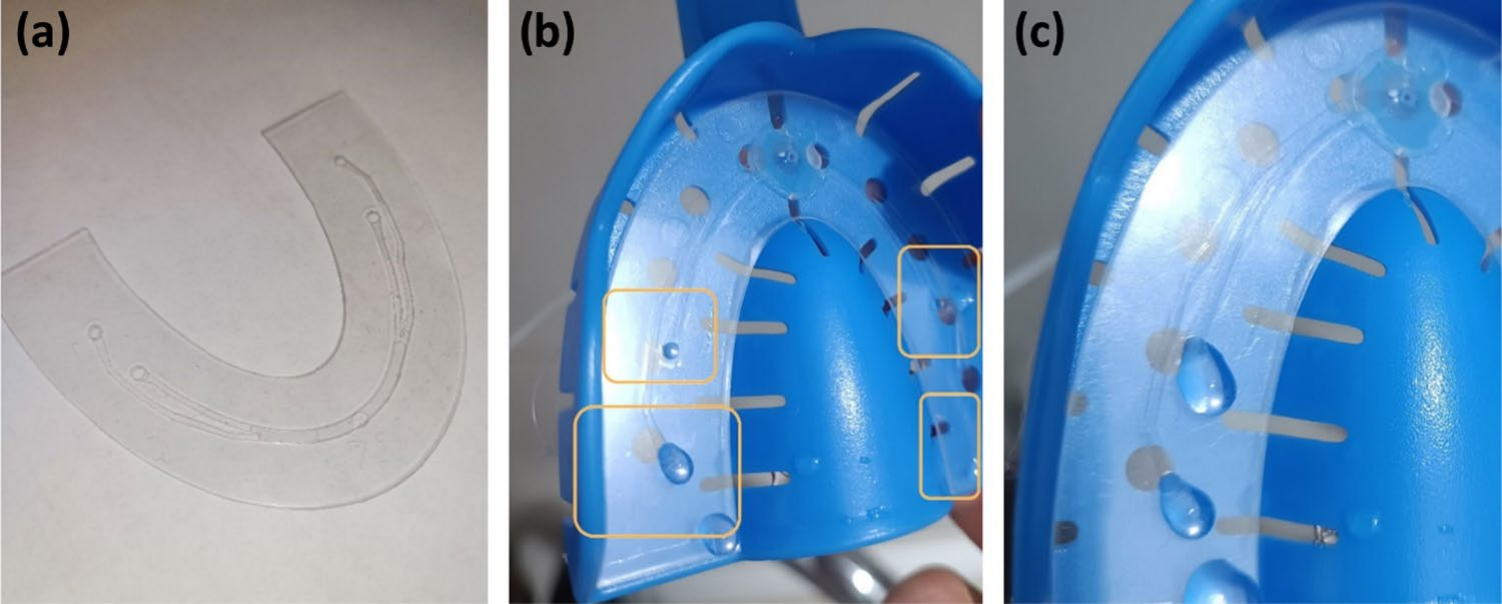
(**a**) Fabricated microfluidic chip, (**b**) testing phase in dentistry spoon holder, (**c**) zoomed details of delivering drops of fluid on exact teeth number 5 and 6 (for demonstration).

Figure 6 illustrates the components of a fabricated microfluidic chip (Fig. 6a) and testing results that depict liquid dispensing from each microfluidic outlet (Fig. 6b,c).

The IOPM theranostic device platform has been tested successfully for DI and AS fluids delivery efficiency and temperature-dependent reaction time for liquid dispensing. Table 1 shows the descriptive data analysis for the entire liquid dispensing sample tested. Mean values with standard deviations for the volume of the *Delivered Liquid* and the *Dead Volume* of the whole system. The normality of distribution was tested using the Shapiro–Wilk test. Since the data showed the normal distribution, parametric methods and Student t-tests were employed for the data analysis.

**Table 1:** Descriptive statistics for the delivered liquid and dead volume for DI and AS.

Descriptive	Delivered liquid (mL)		Dead volume (mL)	
	DI	AS	DI	AS
Mean	608	234	303	212
Median	609	206	321	207
Standard deviation	17.8	73.6	77.2	87.2
Minimum	583	143	160	107
Maximum	634	412	395	360
Shapiro–Wilk W	0.934	0.859	0.93	0.912
Shapiro–Wilk p	0.45	0.055	0.407	0.256

**Figure 7:**
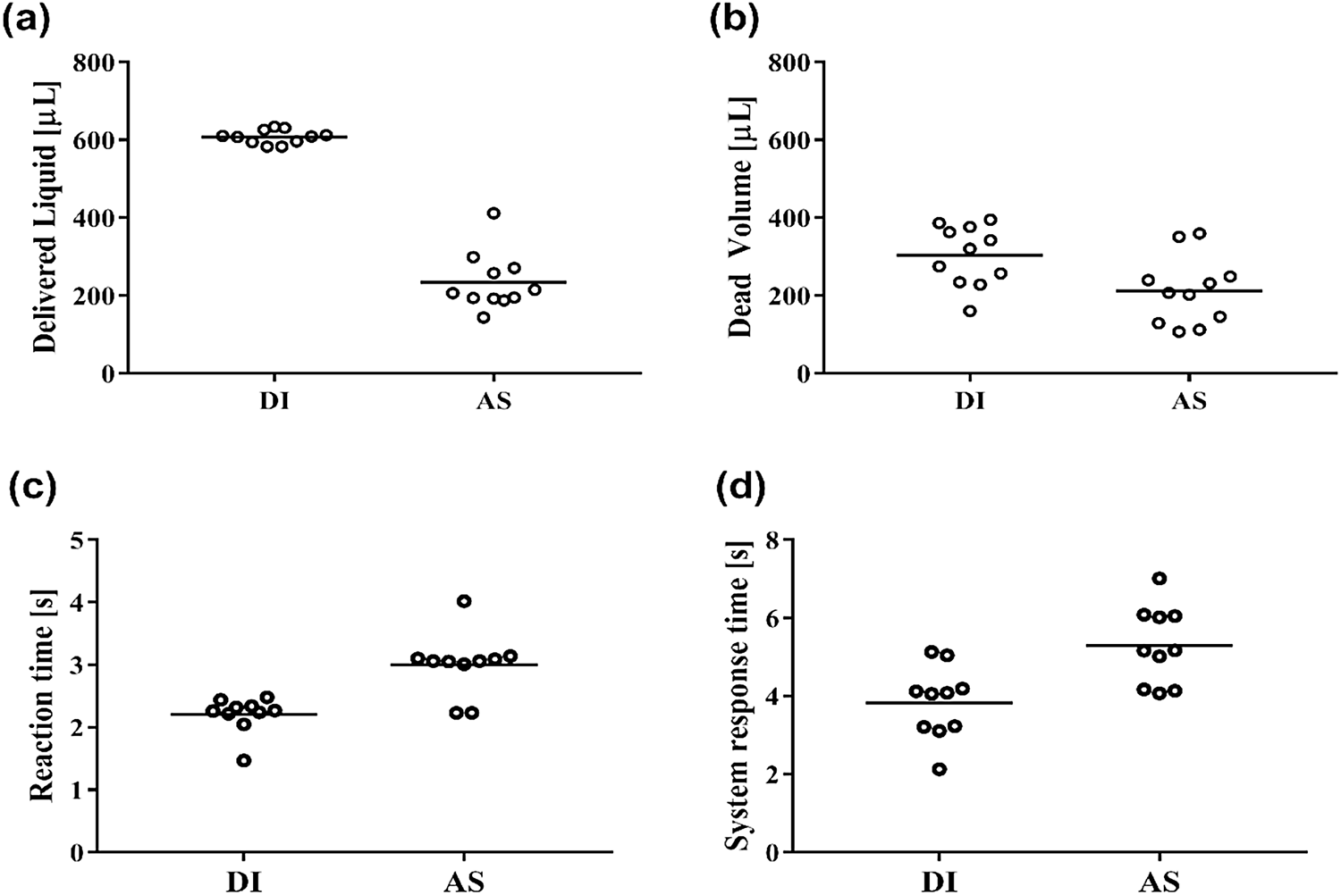
(**a**) *Delivered Liquid*, (**b**) *Dead Volume*, (**c**) temperature reaction time, (**d**) system response time for DI and AS.

The experimental results demonstrated a significant difference when using DI water and AS for the dead volume, as shown in Table 1 and Fig. 7a. The difference is reflected in the amount of *Delivered Liquid,* which differs almost three times. We expected such results considering these two liquids’ different viscosities and surface tension. In a Newtonian fluid, the flow rate in cylindrical tubing decreases as viscosity increases according to Poiseuille’s law^36^. Hence, for the same parameters (micropump settings, duration of discharge), we would get a reduced release of liquid using AS, which was also demonstrable by measurement.

Table 2 and corresponding to Fig. 7a,b show the values of the comparative analysis of two volumes (the *Delivered Liquid* and the *Dead Volume*) of both examined fluids (Student’s t-test), where *Delivered Liquid*-DI and *Delivered Liquid*-AS, as well as *Dead Volume*-DI and *Dead Volume*-AS were compared. Results showed that the obtained values are statistically significant for tested liquids and all tested parameters (p < 0.05, Student’s t-test). Dead volume represents fluidic retained inside the microfluidic chip and tubing.

**Table 2:** Post hoc pairwise analysis.

Parameter	Paired samples T-test			Statistic	df	p
*Delivered Liquid*	DI	AS	Student’s t	15.8	10	< 0.001
*Dead Volume*	DI	AS	Student’s t	3.31	10	0.008

The recorded measurement represents the volume of fluid released on dentures in the practical application of this device is presented in Fig. 7a. The *Dead Volume* (remaining fluid in the chamber) was also measured and is shown in Fig. 7b.

In order to determine-temperature dependent system reaction time, we measured the reaction time from the beginning of the temperature change until the start of the system (when the first droplet exits the system). The temperature reaction time is shown in Fig. 7c. In the case of DI water, the system reaction time was statistically significantly shorter (p < 0.05, Mann–Whitney test).

Furthermore, we measured the system response time–time needed to start the system from an empty system (system full of air) until the start of the system (system full of liquid)—when the first droplet shows at the microfluidic outlets (Fig. 6b). These system response times are depicted in Fig. 7d. Similarly, for DI water, the system response time was statistically significantly shorter (p < 0.05, Mann–Whitney test).

The studies analyzing intraoral temperature have yielded several significant findings. There are reports in the literature that, for healthy adults, the optimal range for intraoral temperature falls between 36.8 and 37.0 °C. At the same time, intraoral temperature fluctuations were investigated with a focus on inter-racial variation and association with outside temperature^37^. The temperatures measured at the incisor and premolar had median values of 34.9 °C and 35.6 °C, respectively, and ranged from 5.6 to 58.5 °C and 7.9 to 54 °C, respectively. There was just a weak correlation between intraoral temperatures and ambient temperature. This study also discovered that the temperature distributions between the Asian and Caucasian groups were significantly different. During the 24-h period, temperatures at the incisor site were in the range of 33–37 °C for around 79% of the time, and 92% of the time at the premolar site. But, when it comes to equally important intraoral underlying structures, research has indicated that on average, the temperature of the alveolar bone is about 5 degrees Celsius lower than that of the mucosa covering it^38^. Temperature variations have been observed in many physiological and pathological conditions. For example, it has been reported for gingival lesions, generally being warmer than the gingival crest, while chronic lesions tend to exhibit lower temperatures^39^. But depending on the environmental influences, some reports go in line with the finding that the physiological temperature range of 20–60 °C^40^. When it comes to pathological conditions, it has been reported that acute lesions were found to be warmer than the gingival crest, while chronic lesions generally exhibited cooler temperatures^41^. These findings emphasise the importance of maintaining an appropriate range of intraoral temperature for overall oral health and comfort and relevance of our developed device to efficiently monitor the temperature and the temperature changes within the above mentioned clinically relevant temperature range, and detect the temperature changes.

There is a limited amount of information available in the literature regarding temperature sensors designed for use inside the mouth. They are made with the intention of precisely measuring the temperature of the mouth cavity. In one method, temperature measuring electrodes are provided in a flexible portion of the catheter shaft and a catheter is equipped with temperature sensors. Temperature sensors are installed into conductive columnar bodies and connected to the electrodes used to detect temperature through these electrodes^42^.

Wearable intraoral sensors have been created with a wide range of characteristics and capabilities. These sensors utilize small strain gauge sensors to measure the forces exerted by the tongue and lips, and they employ an integrated circuit (IC) sensor interface to manage multiple sensors concurrently. In addition, these sensors incorporate a low-energy transmission module to process signals and wirelessly transmit data using the Bluetooth® Low Energy protocol. The design of these sensors places importance on correcting output errors caused by initial strain in real-time, as well as achieving compact dimensions and wireless data transmission, thereby eliminating the necessity for external cables. Digital intraoral sensor designs differ across various models, showing variations in cable arrangements, connectivity interfaces, the inclusion of radiation shields for back-scattering, plate thickness, and the active sensor area. Nonetheless, the industry lacks standardization, and manufacturers often do not readily disclose specific details about the physical design^43^. Table 3 summarises the key findings and comparison of the temperature measurement in oral cavity by researchers.

**Table 3:** Summary of key findings and comparison of temperature measurement in oral cavity.

References	Key findings	Comparison with previous studies	Novelty in the presented research
Volchansky^38^	Average temperature of the healthy oral mucosa was discovered to be 5 °C lower than the temperature of the underlying bone	The temperature was measured using both digital thermometers and fine thermocouples, with the latter being calibrated against precise mercury thermometers to guarantee accuracy	Temperature sensor integrated into the device for oral use Ability to measure temperature in different areas of the oral cavity
Mackowiak^44^	37.0 °C (98.6 °F) and 36.8 °C (98.2°F) are within the range of normal oral temperatures for healthy adults The “normal” oral temperature is a range of temperatures throughout the day	Determination of the average range Defining the “normal temperature	The possibility of the continuous monitoring Wearability Possibility of liquid delivery
Van den Bruel^45^	Mean difference between Tempa Dot and mercury thermometer: 0.04 °C—Sensitivity and specificity for Tempa Dot: 79.0%	Comparison with mercury thermometer The use of thermometer orally and rectally	The use of calibrated sensors with extended and modified wide intraoral modification Possibilities of data collection
Ruan^42^	Measured temperature coefficient: 0.2375 nm/℃—Temperature resolution: < 0.1℃	Proper long-period fiber grating (LPFG) design—Coating LPFG with temperature-sensitive thin film	Proof of concept for intraoral diagnostic platform that combines intraoral drug delivery
Maity et al.^46^	The design involves a spatiotemporal sensing arrangement using graphene-ink printed layers on cellulose networks for real-time molecular analysis in biomedical applications	Spatiotemporal nano-/micro-structural arrangement enables real-time molecular analysis Hierarchically stacked geometrical configuration provides mass spectrogram	Use of nonspecific biomarkers for health monitoring (temperature and humidity) Integration of microfluidics, sensing, drug delivery and intraoral therapeutical devices
Chen^47^	Humidity sensors in medicine are used in respiratory equipment, sterilizers, incubators, pharmaceutical processing, and biological products	Capacitive structures with porous humidity-sensing materials Hybrid dielectric materials with atomic layer deposition	Sensor integrated within intraoral device Connection of the sensor and activation of the liquid delivery

The design of a wearable intraoral diagnostic platform is a promising field in biomedical engineering. It involves integrating a powerful system-on-chip processor, a smart power management unit, and multi-function peripherals into a miniature module. Some wearable intraoral diagnostics use a spatiotemporal nano-/microstructural arrangement, consisting of hierarchically stacked geometrical configuration based free-standing films formed of porous, fibrous, and naturally helical cellulose networks. These films have functionalized hybrid spin-sensitive graphene-ink printed sensing layers that are scalable, binder-free, and printed without a binder^46^. The HSGC’s time–space-resolved architecture produces a mass spectrogram, allowing for the separation, elution, and detection of distinct molecules within mixtures. This architecture offers real-time and wide-spectrum molecular analysis for various biomedical applications, such as wearable spectrometry and quick on-site biopsy decision-making for surgical oncology^48^.

Humidity sensors have various applications in dentistry and clinical medicine. In healthcare settings, they regulate humidity for optimal conditions. Organic substances, like molecular rectifiers, offer precise humidity data^43^. Metal–organic frameworks and their derivatives also exhibit great potential as materials for humidity sensing, primarily due to their remarkable porosity and stability^49^. Moreover, the development of printed capacitive humidity sensors, featuring stacked parallel-plate electrodes, has yielded sensors that possess high capacitance and sensitivity, all while maintaining an economical cost. Furthermore, substrates coated with moisture-sensitive substances can be employed to gauge moisture levels by detecting alterations in light output^6^. In healthcare, humidity sensors in mobile apps monitor patients’ conditions. These apps save data in the cloud for analysis by medical professionals or caregivers^50^.

Intraoral humidity can be measured via various methods, such as using an intraoral moisture meter or measuring breath humidity. Dentine adhesion studies have also investigated the influence of relative air humidity and temperature on moisture in the oral cavity, considering factors such as the use of a rubber dam and nose/mouth breathing^51^.

Exaclear GC, a widely used clear vinyl polysiloxane, is extensively employed in the technique of Injection Moulding for a variety of purposes. Its application is commonly observed in aesthetic and wear cases, as well as in temporary crown and bridge work. Furthermore, Exaclear GC is highly appropriate for the layering of composite materials, particularly when light-curing is necessary from the palatal side. Moreover, its efficacy extends to bite registration and the facilitation of the transfer of brackets and fibers from the model to the oral cavity^52–54^. Vinyl polysiloxane compounds have a significant impact on the field of dentistry due to their extensive range of applications, exceptional biocompatibility, and adherence to high safety standards. These compounds are frequently utilized in the creation of dental impressions, which serve as an accurate replication of the oral tissues for various restorative procedures such as crowns, bridges, and dentures. By offering superior detail reproduction and dimensional stability, VPS materials guarantee the precise fitting of prosthetic restorations. Additionally, these materials have proven to be safe for intraoral usage as they possess low toxicity, limited allergy potential, and excellent tissue compatibility. The hydrophobic nature of vinyl polysiloxane materials also plays a crucial role in effectively managing moisture during the impression-taking process. Consequently, due to their remarkable biocompatibility and safety features, these materials have become a favored option in contemporary dentistry, facilitating successful and dependable outcomes for both patients and dental professionals^55,56^. Previous studies have made attempts to incorporate sensors into vinyl polysiloxane (VPS) devices for various applications. For instance, researchers have explored the adaptation of dental appliances to measure intraoral pressure during functional activities^57^. This innovative approach enables the assessment of pressure changes within the oral cavity, providing valuable insights into occlusal forces and bite dynamics. In another investigation, the performance of a temperature sensor embedded into a mouthguard was examined^13^. This research aimed to evaluate the accuracy and reliability of temperature measurements during oral activities. Additionally, the potential of an oral-based bioguard to estimate heart rate using photoplethysmography has been investigated^58^. By integrating sensors into VPS devices, these studies have demonstrated the versatility and potential of vinyl polysiloxane materials in creating functional and biocompatible dental appliances capable of monitoring physiological parameters.

Currently, intraoral sensors and drug delivery devices use an oral appliance or wearable patch to be placed in the oral cavity for an extended amount of time. For example, France et al.^24^ introduced a system for ultrasound-based drug delivery in the oral cavity to treat inflammatory diseases using delivered therapeutics to mucosal surfaces. However, that system does not contain any sensors. Additionally, the same group of researchers developed a microneedles patch to deliver macromolecules and applied it to the buccal area^25^. On the other hand, Shi et al.^26^ reported a dental patch that can wirelessly, using NFC, observe the oral microenvironment (e.g., pH value) and deliver drugs if it is necessary. The miniature device reported in Ref.^26^ was intended to be mounted on the tooth and may cause discomfort or interfere with the visual and functional appearance of the subject. Our proposed IOPM theranostic device is based on a conventional dental tray which is regularly used in clinical practice, and we have enhanced this tray with embedded sensors and precise fluid-delivering functionalities.

One major advantage of the proposed system is that it is only required to be used when necessary by the user or clinician rather than being worn continuously like previously reported drug delivery systems. This allows for greater flexibility, comfort, and convenience for patients and dentists, as they can use the tray as needed for their treatment or diagnosis and then remove it when finished (similar to taking dental impressions, which is a very common procedure). This could lead to greater patient adherence and satisfaction with the treatment process, as wearable intraoral appliances can cause discomfort. In addition, the microfluidics and device design could be customised for each patient using casting mould for a personalised treatment process targeting certain areas of the denture. An instance of clinical usage is that the smart dental tray could deliver artificial saliva to the denture depending on the oral environment (temperature and humidity) and will be helpful for patients suffering from xerostomia.

One limitation of the system is that the proposed device is not intended for continuous monitoring and delivery; it is designed for on-demand or intermittent use. Currently, this proof-of-concept system has been demonstrated using readily available sensors. In the future, this electronic microfluidic platform can be enhanced by incorporating additional sensors, such as pH sensors and electrochemical biomarker sensors, enabling precise medication delivery to specific locations for personalized diagnosis, treatment, and improved drug administration. Furthermore, the system in its entirety can be further miniaturized and customized to better fit within a mouthguard. This device offers real-time feedback and data for both patients and dentists, potentially enhancing treatment outcomes.

## Conclusions

Measuring important parameters in our oral cavity is of high importance for providing adequate therapy and for maintaining appropriate oral microenvironments. In the current literature, the usual approach was to develop a miniature microdevice that can be attached to a specific tooth. In contrast, we introduce a novel concept that utilizes a widely used dental tray. We have repurposed this tray, traditionally used for dental impressions, by integrating temperature and humidity sensors along with a built-in microfluidic chip for precise fluid delivery. Our research has demonstrated the functionality of this sensor-equipped dental tray in detecting changes within the oral cavity and administering tailored medications using a casted model. This proposed device holds potential value for dental professionals, individuals requiring personalized oral drug delivery solutions, and those who find wearing dental appliances uncomfortable. In future, we aim to expand this platform to encompass multiple biosensors, enhancing safety and precision in personalized drug delivery for therapeutic purposes.

## Data Availability

A summary of the experiment results is enclosed in the manuscript. Additional supporting information is available upon reasonable request to corresponding author, Fatimah Ibrahim.
